# Effect of Different Standard Geometry Shapes on the Tensile Properties of 3D-Printed Polymer

**DOI:** 10.3390/polym15143029

**Published:** 2023-07-13

**Authors:** Rawabe Fatima Faidallah, Muammel M. Hanon, Varun Vashist, Ahmad Habib, Zoltán Szakál, István Oldal

**Affiliations:** 1 Mechanical Engineering Doctoral School, Szent István Campus, MATE University, Páter Károly u. 1, 2100 Gödöllő, Hungary; faidallah.rawabe.fatima.2@phd.uni-mate.hu (R.F.F.); naveenk16@yahoo.co.in (V.V.); 2 Baquba Technical Institute, Middle Technical University (MTU), Muasker Al-Rashid Street, Baghdad 10074, Iraq; 3 Department of Power Engineering, Aleppo University, Myrdian Street, Aleppo 999, Syria; a.habib@alepuniv.edu.sy; 4 Institute of Technology, Szent István Campus, MATE University, Páter Károly u. 1, 2100 Gödöllő, Hungary; szakal.zoltan@uni-mate.hu (Z.S.); oldal.istvan@uni-mate.hu (I.O.)

**Keywords:** fused deposition modeling (FDM), PETG, different geometry shapes, tensile properties, mechanical characteristics, print orientation

## Abstract

This study presents a comparative analysis of the tensile properties of 3D-printed polymer specimens with different standard geometry shapes. The objective is to assess the influence of printing orientation and geometry on the mechanical performance. Rectangular-shaped ASTM D3039 specimens with angles of 0°, 15°, and 90° are compared to various tensile test specimens based on ASTM and ISO standards. All specimens are fabricated using polyethylene terephthalate glycol (PETG) material through fused deposition modeling (FDM). Two printing orientations, flat and on-edge, are investigated, and tensile strength, elastic modulus, strain, and elongation at break are measured. The study examines the weak spot commonly found at the neck of the specimens and evaluates the broken areas. Additionally, a numerical analysis using the finite element method (FEM) is performed to identify stress risers’ locations in each specimen type. Experimental results show that the ASTM D3039-0° specimen printed in the on-edge orientation exhibits the highest tensile properties, while the flat orientation yields the best results in terms of the broken area. The ISO 527-2 specimens consistently display lower tensile properties, irrespective of the printing orientation. The study highlights the enhanced tensile properties achieved with the rectangular shape. Specifically, the tensile strength of ASTM D3039-0° was 17.87% and 21% higher than that of the ISO 527 geometry shape for the flat and on-edge orientations, respectively. The numerical analysis indicated that the ISO 527-2 specimen had either no or minimal stress raisers, and the higher stresses observed in the narrow section were isolated from the gripping location. The findings contribute to understanding the relationship between standard geometry shapes, printing orientation, and the resulting tensile properties of 3D-printed polymer specimens.

## 1. Introduction

The advent of additive manufacturing (AM), also known as 3D printing, during the third industrial revolution brought about a new method of producing components [1]. Stereolithography and fused deposition modelling (FDM) were among the earliest techniques used. FDM gained popularity as a fast prototyping method, utilizing a heated nozzle to melt thermoplastic filament and deposit it layer by layer [2]. During the process, ultrafine and fine particles and vapors are generated as byproducts [3]. The properties and finishing quality of AM objects are influenced by material chemistry and process parameters [4,5]. The literature has extensively reviewed the impact of processing parameters on dimensional accuracy, mechanical properties (including tensile [6], fatigue, and compressive behavior), tribological characteristics [7], and surface quality [8]. Parameters such as filling percentage [9], layer height [10], infill pattern [11], build orientation [12], extrusion temperature [13], and contour width [14] have been widely discussed. Various thermoplastic materials, including acrylonitrile butadiene styrene (ABS), polylactic acid (PLA), polyethylene terephthalate-glycol (PETG), polyamide, and polyether ether ketone (PEEK), have been investigated using these parameters. PETG, a saturated thermoplastic polyester, offers superior mechanical, thermal, and strength properties compared to other polymers [15,16], making it a promising candidate for applications requiring shape memory capabilities and excellent printability [17,18].

The tensile strength of a material is crucial as it determines its ability to resist tension forces. Understanding tensile cracking and failure is important, due to its significant influence on mechanical properties. For instance, improving layer and raster thickness can enhance the tensile strength of ABS polymer [19]. Tensile testing enables the comparison of different FDM materials [20] and variations in polymer characterization techniques [21]. Anisotropy and mechanical properties are influenced by process parameters such as print orientation, raster pattern, and dimensions of the tensile specimen [22]. Infill patterns, which affect material behavior due to an inner substructure, have been studied in polylactic acid (PLA) parts [23,24,25]. Interlocking mechanisms resulting from printing parameters, including raster angle, raster width, and contour width, are also under investigation [23,24,26,27]. Studies have examined the effects of layer thickness, build orientation, and feed rate on 3D-printed PLA samples [28], as well as the impacts of raster angle and layer thickness on both PLA and ABS materials [29].

There are two major organizations involved in the field of additive manufacturing (AM), namely ASTM and ISO. The ASTM committee F42, established in 2009, aims to advance knowledge, foster research, and promote the adoption of additive manufacturing technologies by developing industry standards. On the other hand, the ISO technical committee ISO/TC 261 [30], operating since 2011, is dedicated to standardizing various aspects of additive manufacturing. This includes processes, terminology, definitions, process chains (both hardware and software), testing procedures, quality parameters, supply agreements, and foundational concepts [30]. Both ASTM and ISO are actively involved in the development of standards for mechanical testing of additive manufacturing (AM) materials and components. In their efforts, they evaluate the suitability of existing standards for conducting mechanical tests on polymer-based AM materials and parts. The standards are categorized into two groups: one for plastics, which include ASTM D638 [31] and ISO 527-2 [32], and the other for composites, which include ASTM D3039 [31] and ISO 527-4 [33,34]. Apart from the standard recommendations, practical considerations can also influence the decision to conduct customized tests, instead of relying solely on standardized ones, including the selection of specimen type. Specific practical needs drive these choices, as seen in cases where limited feedstock availability or research investigations of new formulations necessitate the use of non-standard subsize specimens [35]. Additionally, for costly materials, smaller samples than the prescribed size may be preferred, due to the destructive nature of tensile testing [35].

The evaluation of tensile properties in FDM parts often relies on dumbbell-shaped specimens, known as “dog-bones”, based on ASTM D638 [36] and ISO 527-2 [32] standards. These standards define the specimen geometry based on the sample thickness or composite type and provide information on various properties [37,38,39]. However, failure outside the intended narrow section is commonly observed, due to challenges in reproducing the dog-bone geometry through FDM printing [24,37,39,40,41,42]. The original purpose of the ample fillet in the narrow section was to reduce stress concentration, but reproducing its curvature poses difficulties in FDM. This can result in structural defects, including abrupt raster terminations, material gaps, and changes in deposition path, leading to abnormal stress peaks and non-axial stress states, particularly in thin specimens [39,43,44,45]. The tensile characteristics of materials have been evaluated using ISO 527-2 and ASTM D638 standards [24,43,46]. However, premature failure of specimens was observed due to stress concentration near the gauge length, caused by extreme shear in the dog-bone’s radius. To address these challenges in FDM printing, an alternative approach involves modifying the dog-bone geometry by increasing the curvature radius [37,39,47]. The application of ASTM D3039, originally designed for polymer-matrix composites, has been proposed as a solution for evaluating neat polymer parts printed using FDM [43,48,49]. ABS samples printed using fused filament fabrication (FFF), adhering to ASTM D3039, exhibited a higher percentage of samples meeting the acceptable failure standard compared to ASTM D638 dog-bone specimens [42]. The fillet radius was identified as a primary factor causing inconsistent failure in ASTM D638 dog-bones, while the inclusion of an additional inner radius improved the performance of type IV specimens. ASTM D638 type IV overestimated the elastic modulus, while ASTM D3039 overestimated the elastic modulus but underestimated the tensile strength. ASTM D638 type I performed equally as well as ASTM D638 type IV and ASTM D3039 for the elastic modulus and strength [42]. In summary, while tensile standards have not been extensively investigated for additive manufacturing (AM) technologies, they have been identified and presented in Table 1.

The relationship between geometry shapes and properties in additively manufactured parts can provide valuable insights for future applications. However, the influence of specimen shape and size on data reliability has not been extensively explored, leading to challenges in making accurate comparisons across technical reports. In this study, we investigate the effect of different tensile geometry shapes and printing parameters on the tensile properties of PETG test specimens produced using FDM. We examine five different specimen configurations based on ISO 527, ASTM D3039, and ASTM D638 standards, with two build orientations (flat and on-edge). Finite element method (FEM) simulations are also conducted to compare different geometries. This research contributes to the exploration of design freedom in 3D printing and fills a knowledge gap regarding the application of different geometry shapes to PETG’s tensile properties.

## 2. Materials and Methods

### 2.1. Manufacturing of 3D-Printed Specimens

The tensile test samples were fabricated on a commercial 3D-printing machine, “Geetech A20M”, of the FDM type. The working area of the printer is 255 × 255 × 255 mm, which has allowed production of several parts at the same time. To gain a thorough understanding of the mechanical characteristics, the material’s processing parameters could be varied while producing the specimens on the FDM printer. The CAD design, which was generated by the CAD program “Solidworks 2021” and exported as an STL file, underwent changes in process parameters. The modifications were applied using the slicing software Ultimaker Cura 5.2.1. The parameters used for printing the specimens are described in Table 2.

The manufacturing of specimens involved the utilization of polyethylene terephthalate glycol (PETG) filament, specifically of the Filanora brand. The PETG filament is black in color and has a diameter of 1.75 mm. The mechanical properties of the Filanora PETG filament are listed in Table 3.

**Figure 1:**
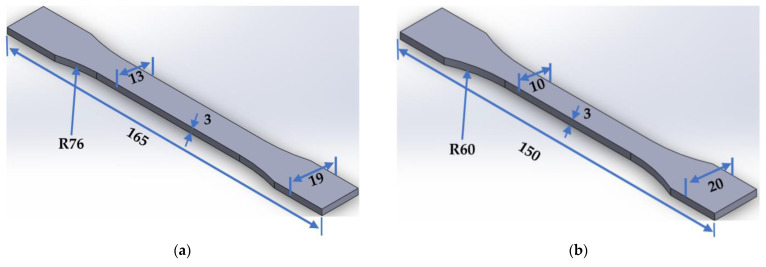

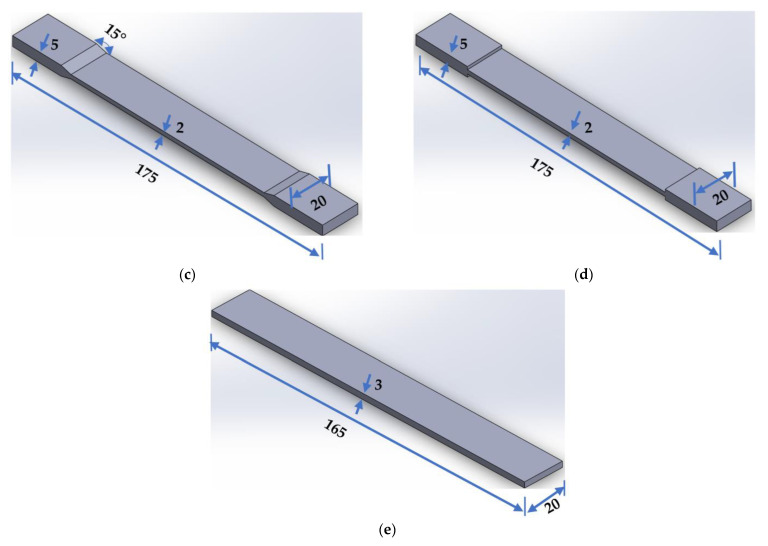
Different geometry shapes of tensile test specimen drawn by SolidWorks software, according to the respective standards: (**a**) ASTM D638, (**b**) ISO 527-2, (**c**) ASTM D3039-15°, (**d**) ASTM D3039-90°, and (**e**) ASTM D3039-0°.

**Figure 2:**
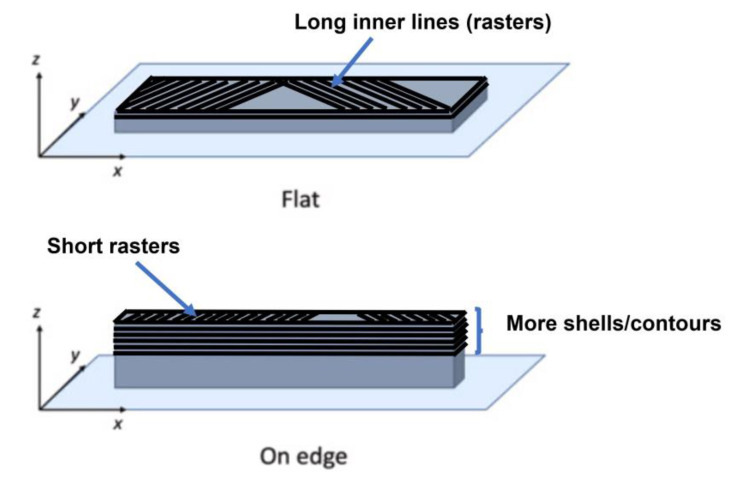
Build orientations examined (flat and on-edge).

Table 4 contains the standards utilized for the manufacturing of the targeted specimens, along with the dimensions and other specifications of each specimen’s type. The tab bevel angles mentioned in the table refer to the tapered termination angles of the end tabs used for the specimens. End tabbing is a technique commonly employed in composite testing to alleviate stress concentrations at the grip edges and promote failures within the gauge section. It involves attaching end tabs to the specimens to distribute the clamping force and protect the specimen surface [50]. Figure 1 illustrates all specimen designs created by using SolidWorks software. All these tensile standard-shape specimens created were printed in two build orientations (flat and on-edge) to determine the effect of print orientation on the mechanical properties as well. Figure 2 illustrates the specimen design orientations used for the production of the tensile testing samples.

### 2.2. Experimental

**Figure 3:**
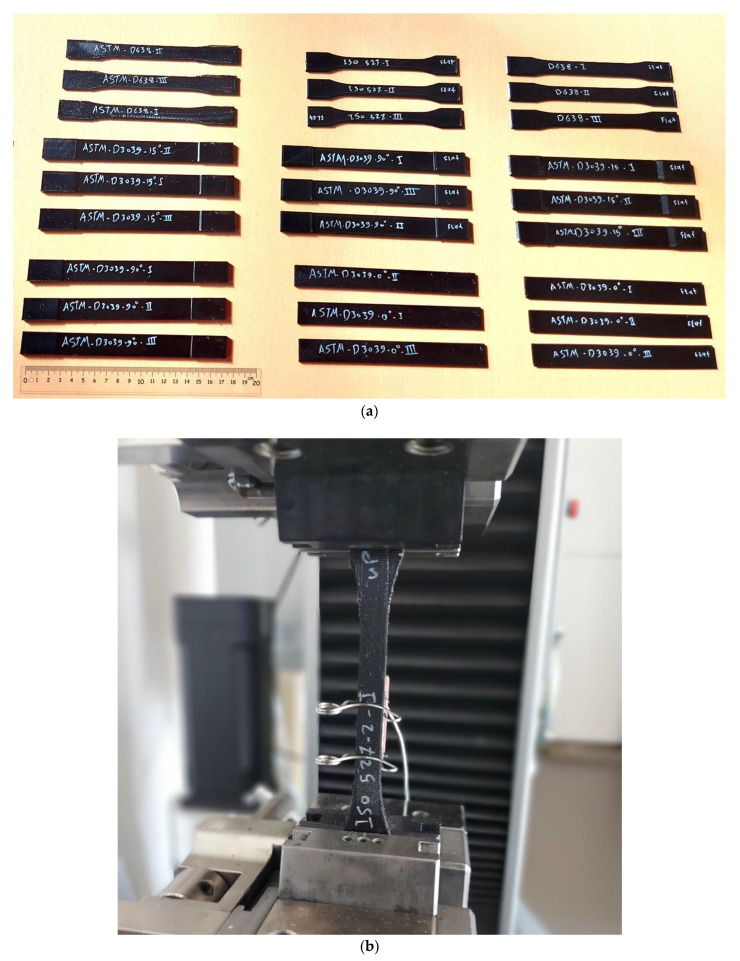
Tensile testing (**a**) sets of tensile specimens of different geometries and (**b**) extensometer attached to the specimen during tensile testing.

The experiments in this study aimed to investigate the influence of tensile test specimens’ geometry on the mechanical characteristics and mode of failure. The Zwick/Roell Z100, a universal testing device, was employed to evaluate the tensile strength of the specimens. Figure 3a illustrates the ten different sets of specimens prepared, originating from five cases of standards with two build orientations, showcasing the diverse geometry shapes investigated. These specimen dimensions adhered to the standards outlined in ASTM D638, ASTM D3039 (0°, 15°, and 90°), and ISO 527-2 for PETG polymer tensile testing.

To ensure robustness and reliability, three identical specimens printed with the same settings were tested from each set under similar conditions. The tests were repeated three times to account for any potential variations. The obtained results were then averaged to obtain representative values. The evaluation of tensile behavior encompassed the determination of tensile strength, tensile Young’s modulus, and the failure mode under the specified conditions.

Notably, Figure 3b highlights the technical importance of the extensometer used in the experimental setup. The extensometer, attached to the tensile test specimens during testing, enables precise measurement of elongation by providing high accuracy in differential movement measurement between two points. With a maximum error of only ±1 µm within the range of 20 to 200 µm, the extensometer significantly contributes to accurate elongation data and facilitates the calculation of Young’s modulus. Moreover, the extensometer measurements allow for the determination of additional mechanical properties, such as nominal strain at tensile strength (εtm), stress at break (σB), strain at break (εB), and nominal strain at break (εtB). 

To conduct the tests, each specimen was securely fixed by the grips, as depicted in Figure 3b, and stretched at a constant speed of 3 mm/min along its longitudinal axis until failure.

### 2.3. Modeling of Tensile Tests by Using Finite Element Method

A series of numerical simulations were performed using the finite element method (FEM) to have a more detailed vision of the effect of specimens’ geometries on the mechanical properties. The commercial software ANSYS 17.2 was used in these simulations.

#### 2.3.1. Modeling

The numerical simulation is an approximation method, where the governing equations are being integrated on every element forming the so-called numerical model. For structural analysis, the so-called weak form was recovered and applied according to Equation (1):

(1) $$\int_{Ω}^{\;}\sigma_{ij}\delta u_{ij}dV=\int_{\partial Ω}^{\;}{\hat{t}}_{j}\delta u_{i}dA$$

This equation is applied to every finite element (Ω), with its closure (∂) as the boundary and the underlying continuum body’s shape. The material was treated as an isotropic material with constant properties in all directions. The isoperimetric Galerkin method [51] selects the test function ($\delta u_{i}$) from the same Hilbertian Sobolev space as the displacement field ($u_{i}$). The system was simplified, and the linear strain measure was utilized because the deformation on a tensile test is minimal. The strain and stress could be calculated using Equations (2) and (3), respectively:

(2) $$\varepsilon_{ij}=\frac{1}{2}\left(u_{ij}+u_{ji}\right)$$

Hooke’s law can be applied because of the observed elasticity without rate effects,

(3) $$\sigma_{ij}=C_{ijkl}\varepsilon_{kl}$$

where the stiffness tensor of rank four ($C_{ijkl}$), the stress tensor ($\sigma_{ij}$), and the strain tensor ($\varepsilon_{ij}$), are linearly connected.

#### 2.3.2. Meshing and Boundary Conditions

**Figure 4:**
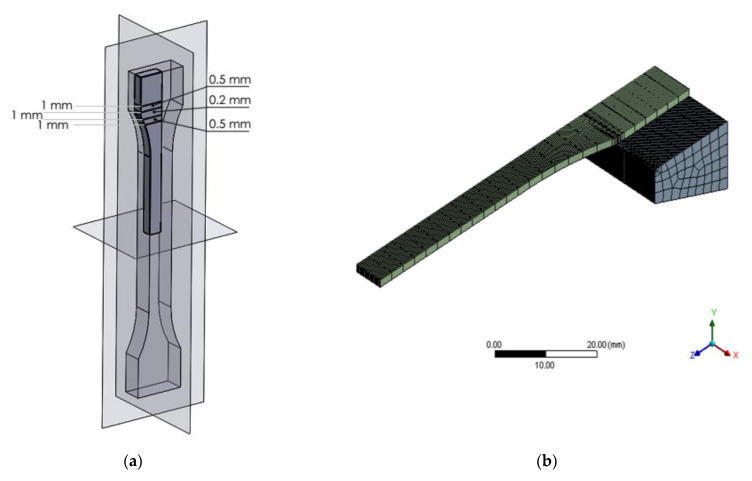
Numerical simulation (**a**) symmetry planes and refinement regions (**b**) the meshing.

In every numerical simulation, the numerical mesh plays a key role in obtaining valid results. In order to benefit the geometrical symmetry and reduce the computational cost, every specimen’s geometrical model was clipped using its symmetry plans, resulting in only one eighth of the original geometry and half of the gripping mechanism from the experimental apparatus. Furthermore, a uniform element size was used, except for the regions with high changing rates in cross-section areas, as shown in Figure 4.

**Figure 5:**
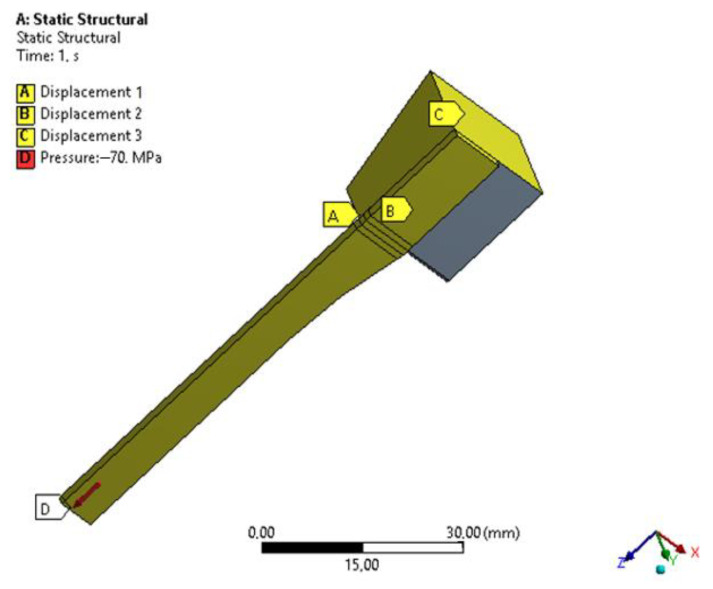
Boundary conditions employed in the simulation.

As for the boundary conditions, to emphasize the true geometrical effect and to exclude the effect of different (Force vs. Elongation) curves, a uniform stress of 70 MPa was applied to every numerical study, with a frictional contact between the specimen and the gripping jaw alongside with a zero displacement of the gripping jaw, as shown in Figure 5.

## 3. Results and Discussion

### 3.1. Experimental Results

Various variables, as discussed in the literature, can effectively increase stiffness and strength. The major variables that influence deformations and deflections include material properties, layer binding, and FDM 3D printing parameters (mainly, infill pattern and build orientation). To delve deeper into the characteristics of build orientations, it is helpful to look at the structure of the printed tensile test specimens in Figure 2. Every layer has inner lines and a shell (contour). The direction of the layer contour for the flat sample is parallel to the applied tensile test force. These specimens exhibited a higher likelihood of elongation, due to the presence of long internal lines constructed at a 45° angle and a sufficient number of layers, resulting in increased strain. The on-edge workpiece possesses a complex structure, characterized by a cross-section with numerous layers, a narrow contour, and short internal lines. This explains how these samples were pulled with a great degree of strength during the test.

**Figure 6:**
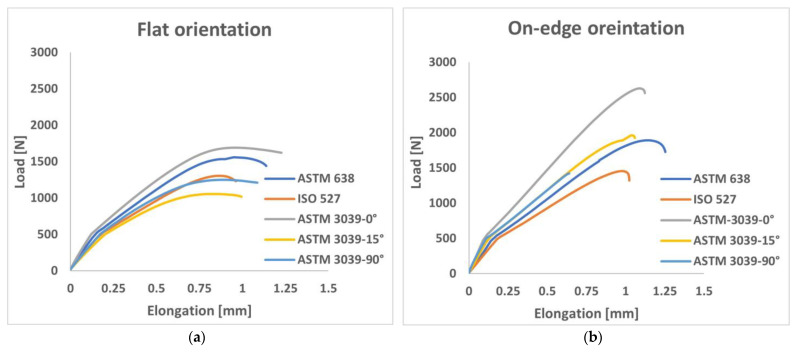
Load-displacement curves for (**a**) flat build orientation and (**b**) on-edge orientation.

The load-displacement curves that were obtained from the tensile test for all examined shape geometry and orientations are shown in Figure 6. It is clear that the build orientation of the 3D printing parameter and the shape geometry has a significant impact on the values of force versus elongation. The results were divided into two diagrams according to the build orientations and the shapes of tensile standards examined. The highest applied loads required to reach the fracture, for the different shape geometry, were reported to the ASTM D3039-0° (for both flat and on-edge), as shown in Figure 6. The average values (out of three testing results for each orientation) of these highest loads obtained were 1693 N and 2632 N for the flat and on-edge orientations, respectively. Comparably, ASTM D638 standard specimens also showed a relatively high load, ranging between 1562 N and 1890 N for the flat and on-edge orientations, respectively. In contrast, the lowest loads were reported in specimens of the ASTM D3039-15° (at the flat build orientation) and ISO 527 (at the on-edge build orientation), with values of 1026 N and 1475 N, consecutively.

**Figure 7:**
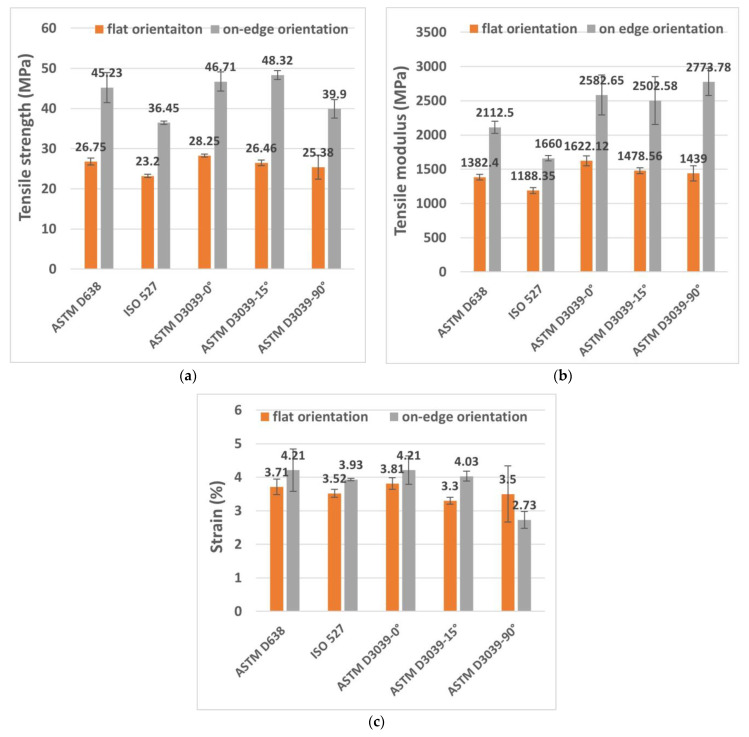
Results of different geometry shape specimens during tensile test for flat and on-edge build orientations’ (**a**) tensile strength, (**b**) tensile modulus, and (**c**) tensile strain.

Figure 7a–c presents the results of the ultimate tensile strength, tensile modulus, and tensile strain of the tested specimens. In terms of the tensile strength, it can be seen from Figure 7a that, at flat build orientation samples, the values ranged between 23.2 MPa and 28.25 MPa, where the best strength was from ASTM D3039-0°. However, the on-edge build orientation showed higher tensile strength, ranged between 36.45 and 48.32 MPa, and the better values were from ASTM D3039-15°. In both flat and on-edge orientations, the lowest tensile strengths were from the ISO 527 specimens. The increased tensile strength of these specimens is due to their reliable geometry shape, which avoided the weak spot at the neck (curvature) of the specimens of ASTM D638 and ISO 527. Therefore, the applied load was distributed across a larger area, resulting in higher resistance to failure. Regarding the effect of print orientation, generally, the on-edge specimens revealed much higher tensile strength as compared to the flat-printed ones. For instance, the average of the ASTM D3039-0° tensile strength for the on-edge orientation was 39.52% higher than that of the flat orientation. Furthermore, the increased tensile strength of ASTM D3039-0° for the flat and on-edge orientations was higher than the ISO 527 geometry shape (the weakest) by 17.87% and 21%, respectively. In a comparison between ISO 527 and ASTM D638, the tensile strength average of ASTM D638 specimens was higher than the average of ISO 527 specimens by 13.27% and 19.5% for the flat and on-edge orientations, respectively.

In terms of the tensile modulus, the flat build orientation values were ranged between 1188 MPa and 1622 MPa, where the best values were from ASTM 3039-0°. On the other hand, the tensile modulus of the on-edge build orientation ranged between 1660 MPa and 2773 MPa, and the better values were from ASTM D3039-90° (see Figure 7b). In addition, the tensile modulus of ASTM D3039-0° was 26.74% and 40.15% higher than the ISO 527 geometry shape (the lowest modulus) for the flat and on-edge orientations, respectively. In a comparison between ASTM D638 and ISO 527, the average of the ASTM D638 specimens was 14% and 21% higher than the ISO 527 geometry shape for the flat and on-edge orientations, consecutively. Concerning the tensile strain (see Figure 7c), which was determined at the yield strength, its values were ranged between 2.73% and 4.21% (for both orientations). The ASTM D3039-0° and ASTM D638 specimens exhibited the highest values, while the ISO 527 specimen showed the lowest value. The tensile strain average value of the ASTM 3039-0° at on-edge orientation was 9.5% higher than the flat orientation of the same shape.

Table 5 presents the average values of various tensile test mechanical properties for each geometry shape. The results demonstrate notable variations in the mechanical behavior of the specimens. For εtm (nominal strain at tensile strength), the ASTM D3039-90° specimen in the flat orientation exhibited the lowest value of 3.7%, while the ASTM-D638 specimen in the on-edge orientation showed the highest value of 5.9%. In terms of σb (stress at break), the ISO 527 specimen with flat build orientation displayed the lowest value of 13.4 MPa, whereas the ASTM D3039-15° specimen in the on-edge build orientation achieved the highest value of 48.3 MPa. Additionally, the ASTM D638 specimens in the on-edge build orientation demonstrated the highest εb (strain at break) and εtb (nominal strain at break) values of 9.5% and 11.2%, respectively.

By testing FDM dog-bone shaped specimens, Kay [52] credited lower tensile strengths and a higher degree of disassembling to the use of ASTM D638, which is not recommended (according to him). The researcher highlighted how failures frequently happened in the neck area of dog-bones manufactured with FDM, as a result of stress concentration in the part’s transition zones. Nevertheless, notable enhancements were observed when conducting tests on parts in accordance with ASTM D3039. It is important to note that ASTM D638 is primarily intended for testing conventional polymeric bulk materials, while the additive manufacturing parts in question more closely resemble composite structures [52]. Conversely, ASTM D3039 is specifically tailored for evaluating composite materials based on their geometry. Consequently, it is anticipated that these parts would exhibit higher values for ultimate tensile strength and modulus, due to a greater proportion of continuous polymer extruded fibers spanning the length of the gauge section.

**Figure 8:**
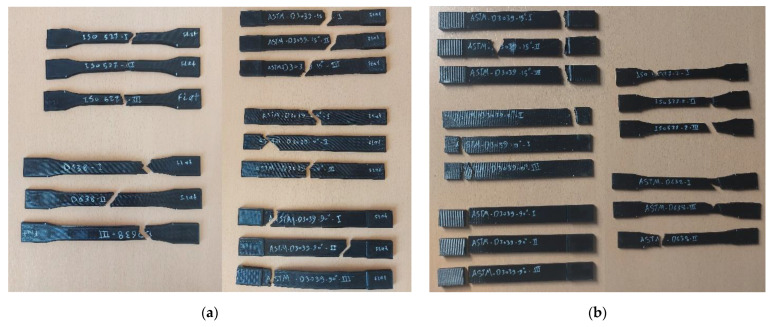
Specimens’ broken area: (**a**) flat orientation and (**b**) on-edge orientation.

Figure 8 shows the broken specimens after the tensile test. As is well-known, it is much better for the breakage/failure to be in the middle of the gauge section than in other parts of specimen. As is obvious from Figure 8, the broken area for the on-edge orientation was always near the edge of the specimen, with the breaks near the gripping area, which is not recommended. In general, the on-edge orientation of FDM samples tends to yield higher strength, as indicated by the findings from various investigated standards. However, the impact of orientation is particularly notable when considering the ASTM D638 and ISO 527 standards. These observations align with the results reported by Aliheidari et al. [53], supporting the notion that the mechanical properties are directly influenced by the distinctive characteristics of the layered structure, specifically the adhesion between the layers. In contrast, the fracture in most of the flat printed specimens was almost in the middle.

**Figure 9:**
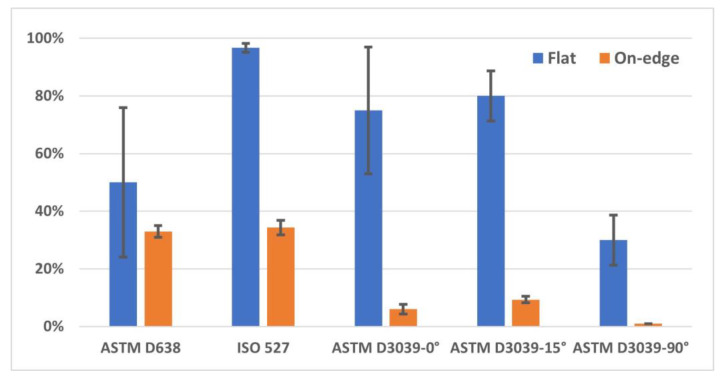
The percentage of good breakage area of specimens.

To distinguish in which geometry shape and printing parameter the specimen’s failure was better, the broken area was given a percentage out of 100% (called the good breakage area) considering the fracture’s placement from the middle of the sample; the closer to the middle, the higher the percentage, and vice versa. Therefore, if the broken area is near the neck/edge, then it is not considered to be a good fracture. Figure 9 shows the percentage of the good breakage area, based on the specimens’ fractures depicted in Figure 8.

**Figure 10:**
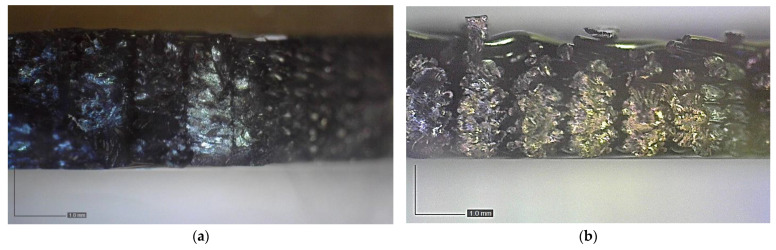

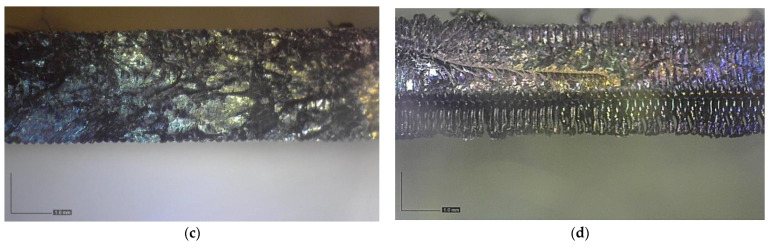
The fracture surface of (**a**) the ASTM D638 specimen in the flat orientation, (**b**) the ASTM D3039 90° specimen in the flat orientation, (**c**) the ASTM D638 specimen in the on-edge orientation, and (**d**) the ASTM D3039 90° specimen in the on-edge orientation.

When examining fracture surfaces of tensile specimens using an optical microscope, the flat and on-edge orientations offer distinct insights into the material’s fracture behavior. The flat orientation allows for a detailed analysis of surface features, crack patterns, and material characteristics, providing valuable information about the material’s response to external forces (see Figure 10a,b). In contrast, the on-edge orientation exposes layered structures, internal defects, and interfacial characteristics, offering a deeper understanding of the material’s internal properties and structural integrity (see Figure 10c,d). By combining these observations, a comprehensive understanding of fracture mechanisms, contributing factors, and the material’s overall response to stress can be achieved. The fracture surfaces observed in both the flat and on-edge orientations exhibit characteristics typically associated with brittle fractures, as demonstrated in Figure 10, which showcase optical microscope images of the fractured specimens. The flat specimen fractured at an angle of 45°, aligning with the structure of its raster direction. On the other hand, the on-edge specimens displayed a fracture angle of 90°. Notably, despite the difference in fracture angles, the on-edge test piece exhibited higher tensile strength, attributed to the robust design of the inner lines and the presence of doubled shell layers. These structural features contribute to improved mechanical properties and enhanced structural integrity, leading to the observed increase in tensile strength.

### 3.2. Numerical Results

In order to ensure the validity of mechanical properties testing, it is important that the geometry of the testing specimen has no influence on its actual mechanical properties. The presence of any stress risers can introduce artificial stress concentrations, leading to inaccurate experimental results. In structural analysis, the stress-strain behavior of a material can be represented using either linear or nonlinear curves. In linear analysis, the material’s stress-strain curve is assumed to be linear, while nonlinear analysis involves implementing a digital representation of the true material stress-strain curve. In this research, both linear and nonlinear modeling methods were employed to evaluate the ability of linear models to accurately capture physical phenomena. For the linear models, a Young’s modulus of 5250 MPa was considered. The nonlinear models incorporated additional material properties, such as a yield strength of 70 MPa and a tangent modulus of 10 MPa. Table 6 presents the maximum Von Mises stress for each specimen, along with the shape-specific stress-increasing effects, using a stress value of 70 MPa in the boundary conditions.

The results presented in Table 6 provide valuable insights into the behavior of the tested specimens. It is evident that the linear stress-strain model tends to overestimate the equivalent stress, while the nonlinear model yields values that are closer to the stress values used in the simulation settings. This discrepancy highlights the importance of using an accurate representation of the material’s stress-strain curve for reliable simulations.

**Figure 11:**
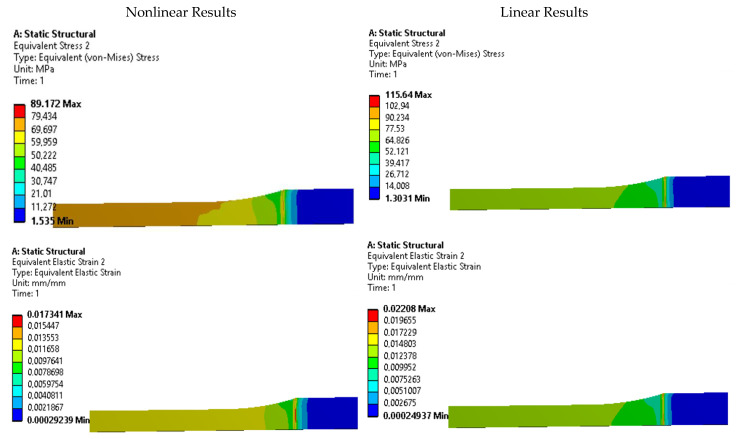
Stress and strain contours for specimen ASTM D638.

**Figure 12:**
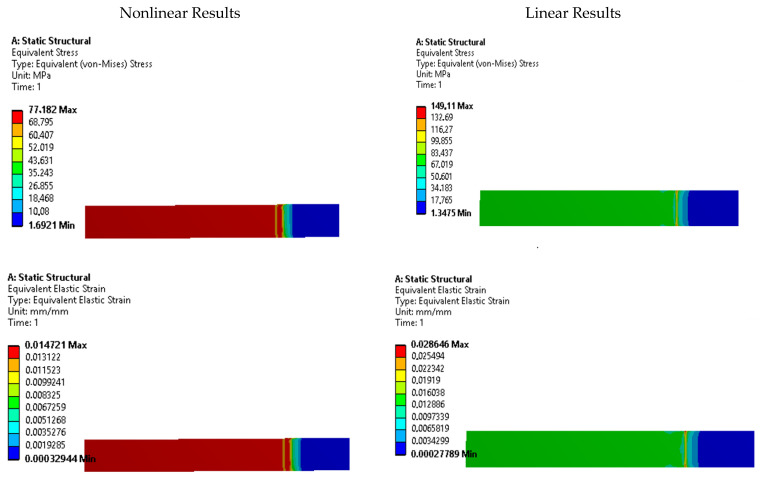
Stress and strain contours for specimen ASTM D3039-0°.

**Figure 13:**
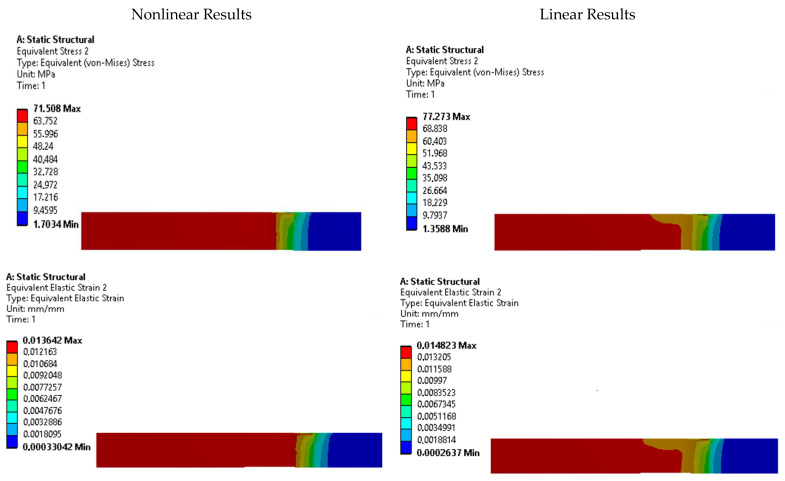
Stress and strain contours for specimen ASTM D3039-15°.

**Figure 14:**
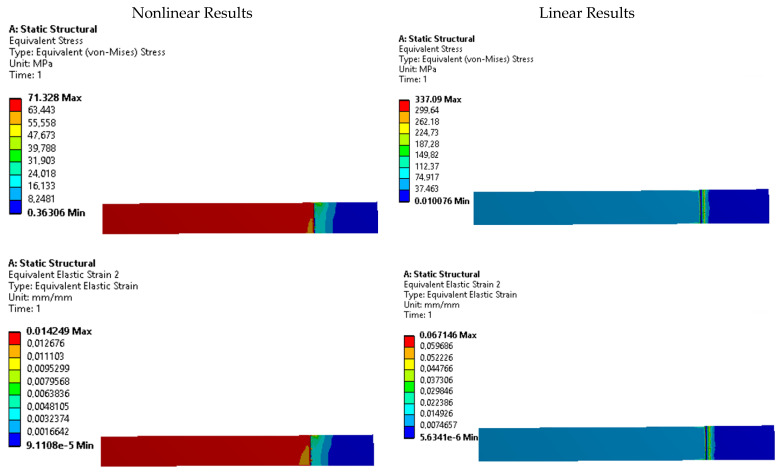
Stress and strain contours for specimen ASTM D3039-90°.

**Figure 15:**
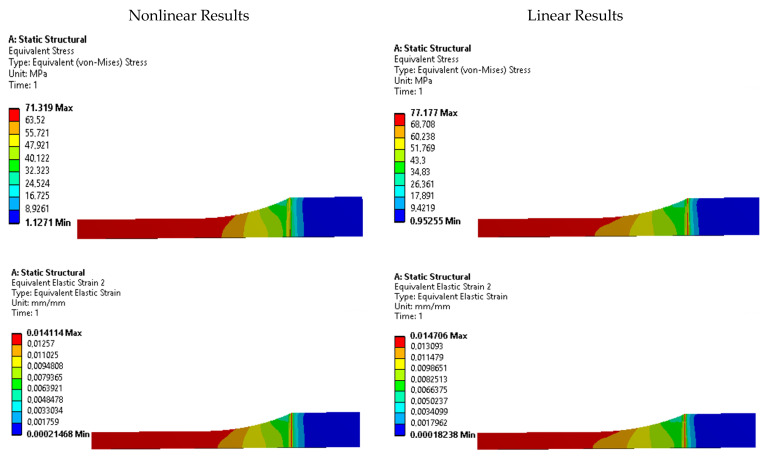
Stress and strain contours for specimen ISO 527-2.

To further investigate the presence of stress risers, contour plots of equivalent Von Mises stress and strain were analyzed for each specimen. Figure 11, Figure 12, Figure 13, Figure 14 and Figure 15 display these contour plots for the ASTM D638, ASTM D3030-0°, ASTM D3039-15°, ASTM D3039-90°, and ISO 527-2 specimens, respectively. Figure 11 reveals a significant stress concentration in the transition area from the gripping location to the narrow middle section, with a maximum stress value of approximately 90 MPa. This value exceeds the applied load during the simulation by 21.5%, indicating the potential for failure or fracture to occur in a location different from the intended narrow section. Such a deviation from the desired fracture location could significantly impact the accuracy and reliability of experimental results.

Similar stress concentration phenomena are observed in the ASTM D3030-0° specimen (Figure 12) and the ASTM D3039-15° specimen (Figure 13), although with reduced intensity. These findings suggest that these specimens may also be prone to inaccurate experimental results due to stress risers. In contrast, the ASTM D3039-90° specimen (Figure 14) and the ISO 527-2 specimen (Figure 15) exhibit minimal or negligible stress risers near the gripping area. The maximum stresses observed in these specimens do not exceed 1.86% of the applied load, and the narrow/gauge section demonstrates a uniform stress distribution consistent with the simulated stress value. This indicates that the ASTM D3039-90° and ISO 527-2 specimens exhibit greater stability during mechanical testing, as they are less affected by the fixture and clamping mechanism compared to the other specimens.

## 4. Conclusions

In this study, the effect of standard specimens’ geometry shapes, manufactured using FDM technology, on the mechanical characterization of polymers has been investigated. Five different geometries were examined for uniaxial tensile studies, utilizing various build orientations (flat and on-edge) specifically for PETG material. The primary aim was to identify the optimal geometry for tensile testing of FDM parts and compare the mechanical performance of different specimen shapes. Additionally, numerical simulations using the finite element method were conducted to identify stress risers in each specimen’s geometry. The obtained results allow for the following observations to be made:

- Preferred specimen geometries: Existing standards recommend dumbbell and rectangular shapes for tensile testing of FDM parts. However, our analysis suggests that rectangular samples with straight edges, such as those conforming to ASTM D3039, exhibit more favorable mechanical characteristics compared to dumbbell-shaped samples with curved edges (ASTM D638). Experimental data indicate that the use of ASTM D3039 rectangular specimens with straight edges reduces the occurrence of stress concentration-induced failures and abrupt transition zones. It is important to note that conflicting results for different geometry shapes may arise due to factors such as feedstock material type, printer configuration, printing parameters, and test procedures.
- Effect of print orientation: The on-edge build orientation specimens demonstrated the best tensile properties, surpassing the flat orientation specimens by 39.4%. This improvement can be attributed to the robust inner structure achieved with the on-edge orientation.
- Numerical analysis of stress concentration: The finite element simulations revealed significant stress concentration in the transition area near the gripping location for specimen types ASTM D638 and ASTM D3039. In contrast, the ISO 527-2 specimens exhibited minimal stress raisers near the gripping area, with higher stresses concentrated in the narrow/gauge section away from the clamping location.

In conclusion, the majority of current standards are appropriate for testing parts made using additive manufacturing (AM). However, additional advice is needed to handle the engineering properties measurements made using AM techniques. Methodologies to evaluate the performance of novel materials and their suitability for particular platforms must be standardized as 3D printing progresses from a tool for prototyping to a mass-production manufacturing technique.

## Figures and Tables

**Table 1:** Different tensile standards used in AM technologies.

Tensile Standard Name	Standard Designation	Description
ISO 527-2-2012 [32]	Determination of tensile properties for plastic. Part 2: Test conditions for extrusion and molding plastics.	Similar to ASTM D638, it is split into five sections, taking into account the various sample types, such as film, isotropic fiber composites, and unidirectional composites.
ISO 527-4:1997 [33]	Determination of tensile properties for fiber-reinforced plastic composite. Part 4: Test conditions for isotropic and orthotropic.	Specific to fiber-reinforced composites. The use of this part may be necessary for specific reinforcements or manufacturing procedures.
ASTM D638 [36]	Standard test method for tensile properties of plastics.	Basic test method to produce tensile properties of plastics. There are several types of dog-bone geometry. Need for high-strength reinforcing.
ASTM D3039 [31]	Standard test method for tensile properties of polymer matrix composite.	The basic test procedure for high modulus fiber composites’ tensile characteristics. Requires a specimen with a rectangular form. Although additive materials do not match reinforcing standards, flaws are reduced by rectangular form.

**Table 2:** The fixed 3D printing process parameters used for manufacturing the specimens.

Parameter	Value	Unit
Layer thickness	0.2	mm
Initial layer height	0.24	mm
Print speed	60	mm/s
Infill speed	30	mm/s
Wall speed	25	mm/s
Printing temperature	230	°C
Building plate temperature	70	°C
Infill density	100	%

**Table 3:** Properties of PETG polymer material used, as provided by the manufacturer.

Properties	Value	Unite of Measure	Standard
Density	1.3	g/cm^3^	ISO 1183
Tensile strength	42	MPa	ISO 527
Tensile modulus	5250	MPa	ISO 527
Elongation at break	7.4	%	ISO 527
Flexural strength	70	MPa	ISO 178
Heat resistance	75	°C	ISO 75

**Table 4:** Standard and specifications of each specimen type manufactured.

Standard	Width of Narrow Section (mm)	Width Overall (mm)	Length Overall (mm)	Thickness of Narrow Section (mm)	Thickness Overall (mm)	Radius of Curvature (mm)	Tab Bevel Angle (°)
ASTM D638	13	19	165	3	3	R76	-
ISO 527-2	10	20	150	3	3	R60	-
ASTM 3039/3039M	20	20	165	3	3	-	0°
ASTM 3039 angle	20	20	175	2	5	-	15°
ASTM 3039 angle	20	20	175	2	5	-	90°

**Table 5:** The average value of εtm, σb, εb, and εtb for each geometry shape examined.

Specimen’s Standard	εtm (%)	σb (MPa)	εb (%)	εtb (%)
ASTM-D638-Flat	4.7	19.5	4.7	5.7
ASTM-D638-On-edge	5.9	19.9	9.5	11.2
ISO 527-Flat	4.6	13.4	4.4	5.6
ISO 527-On-edge	5.6	17.1	7.5	9.2
ASTM-D3039-0°-Flat	4.2	22.79	7	7.5
ASTM-D3039-0°-On-edge	5.23	44.3	4.5	5.2
ASTM-D3039-15°-Flat	3.9	23.3	5.2	5.8
ASTM-D3039-15°-On-edge	5.4	48.3	4	5.4
ASTM-D3039-90°-Flat	3.7	16.8	3.8	4.2
ASTM-D3039-90°-On-edge	4.4	37.9	2.7	4.4

**Table 6:** Von Mises stress values of different tensile geometry specimens.

Specimen	Linear Model		Nonlinear Model	
	Maximum Stress (MPa)	Multiplier Factor %	Maximum Stress (MPa)	Multiplier Factor %
ASTM D638	115.64	39.47	89.17	21.50
ASTM D3039-0°	149.11	53.05	77.18	9.30
ASTM D3039-15°	77.27	9.41	71.51	2.11
ASTM D3039-90°	337.1	79.23	71.33	1.86
ISO 527-2	77.18	9.30	71.32	1.85

## Data Availability

The data used to support this study’s findings can be provided by the corresponding author upon reasonable request.
